# Supplementary material for: Deltamethrin Contact Exposure Mediated Toxicity and Histopathological Aberrations in Tissue Systems of Public Health Importance Cockroach Species Periplaneta americana and Blattella germanica
Source: Front Physiol. 2022 Jul 18;13:926267. doi: 10.3389/fphys.2022.926267 (PMC9340665; doi:10.3389/fphys.2022.926267)
Supplement: Supplementary file 1 [file Table1.docx]

**Supplementary File 1: Corrected Mortality (%) recorded for different concentrations post 1 hr exposure**

|  | **Conc. (%)** | **24hr** | **48hr** | **72 hr** |
| --- | --- | --- | --- | --- |
| **PA** | 0.00025 | 40 | 53 | 70 |
|  | 0.0025 | 64 | 90 | 95 |
|  | 0.025 | 97 | 100 | 100 |
|  | 0.25 | 100 | 100 | 100 |
|  | 0.5 | 100 | 100 | 100 |
|  | 1 | 100 | 100 | 100 |
| **BG** | 0.00025 | 43 | 47 | 70 |
|  | 0.0025 | 63 | 87 | 93 |
|  | 0.025 | 96 | 98 | 100 |
|  | 0.25 | 100 | 100 | 100 |
|  | 0.5 | 100 | 100 | 100 |
|  | 1 | 100 | 100 | 100 |

PA – *P. americana*; BG – *B. germanica*
