# Supplementary figures and images for: Deltamethrin Contact Exposure Mediated Toxicity and Histopathological Aberrations in Tissue Systems of Public Health Importance Cockroach Species Periplaneta americana and Blattella germanica
Source: Front Physiol. 2022 Jul 18;13:926267. doi: 10.3389/fphys.2022.926267 (PMC9340665; doi:10.3389/fphys.2022.926267)

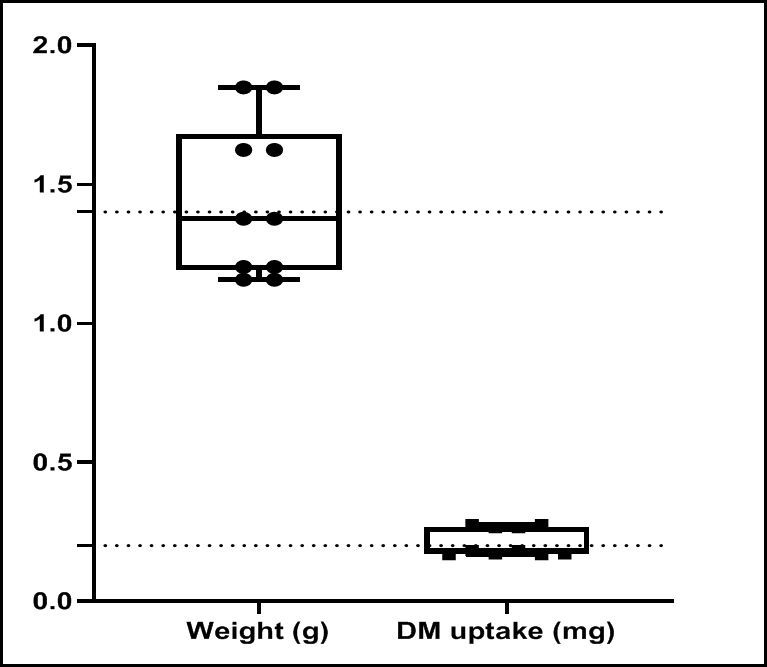

Supplement: Supplementary file 2 [file Image1.jpg]
